# Differential Expression of Plasma Exo-miRNA in Neurodegenerative Diseases by Next-Generation Sequencing

**DOI:** 10.3389/fnins.2020.00438

**Published:** 2020-05-07

**Authors:** Chao Nie, Yuzhe Sun, Hefu Zhen, Mei Guo, Jingyu Ye, Zhili Liu, Yan Yang, Xiuqing Zhang

**Affiliations:** ^1^BGI Education Center, University of Chinese Academy of Sciences, Shenzhen, China; ^2^BGI-Shenzhen, Shenzhen, China; ^3^China National GeneBank, BGI-Shenzhen, Shenzhen, China; ^4^BGI Genomics, BGI-Shenzhen, Shenzhen, China; ^5^Department of Neurology, Affiliated Hospital of Jining Medical University, Shandong, China

**Keywords:** neurodegenerative diseases, exosome, microRNA, AD, PD, biomarker

## Abstract

Neurodegenerative diseases encompass a wide variety of pathological conditions caused by a loss of neurons in the central nervous system (CNS) and are severely debilitating. Exosome contains bio-signatures of great diagnostic and therapeutic value. There is proof that exosomal proteins can be biomarkers for Alzheimer’s disease (AD) and Parkinson’s disease (PD). MicroRNAs in exosome has potential to be an important source of biomarkers for neurodegenerative diseases. Here, we report exosomal microRNA performance of human plasma in neurodegenerative diseases by small RNA sequencing. A wide range of altered exo-miRNA expression levels were detected in both AD and PD patients. Down-regulated miRNAs in AD samples were enriched in ECM-receptor interaction pathway and both up-/down-regulated miRNAs in PD samples were enriched in fatty acid biosynthesis pathway. Compared to the control, 8 miRNAs were found to be significantly elevated/declined in AD and PD samples, of which 4 miRNAs were newly identified. Additionally, two exosome isolating methods were compared and the reproducibility of plasma exo-miRNA expression was confirmed, suggesting the feasibility of large-scale clinical application of this method. This study revealed exo-miRNA expression levels in neurodegenerative diseases, proposed new biomarkers and their potential functional pathway for AD and PD, confirmed the reproductivity of exo-miRNA profiles by using a different exosome isolating method, and compared the results with plasma miRNA expression. Therefore, this study also provides a precedent for identifying exosomal biomarkers of neurodegenerative diseases in plasma by high-throughput sequencing and it could extend the therapeutic repertoire of exosomal biomarkers.

## Introduction

Neurodegenerative diseases are a group of disorders that are characterized by the progressive degeneration of the function and structure of the central nervous system (CNS) (Gao and Hong, 2008). Common neurodegenerative diseases include AD and PD. Traditional diagnosis of neurodegenerative diseases usually uses biomarkers in cerebrospinal fluid (CSF) or positron emission tomography (PET) imaging which are invasive or expensive (McKhann et al., 2011). Using peripheral blood to detect neurodegenerative biomarkers satisfies the rapid and safe requirement of chronic disease prediction (Valadi et al., 2007; Schwarzenbach et al., 2014). Recently, exosome has become an emerging source of biomarkers for various human diseases like neurodegenerative diseases (Alderton, 2015). Exosome, a major component of extracellular vesicle (EV), is a small lipid bilayer particle that is commonly observed in human body fluids involving in cell-to-cell communication and contains the fundamental biological elements such as protein, mRNA, long non-coding RNA (lncRNA), and, especially small RNA (sRNA) (Bellingham et al., 2012). Exosome is a good carrier that can cross blood-brain barrier which blocks most of the signal from the brain (Nelson et al., 2008). Pathological neurons release huge amount of exosome, some of which spread to peripheral blood, and cause the change of exosome-derived contents (Wood et al., 2011). A newly study showed that there is concordance between the assessment of Aβ42, T-tau, and P-T181-tau in CSF and plasma exosomes suggesting that lots of exosomes extracted from human plasma are derived from brain (Jia et al., 2019). Exosomal proteins as biomarkers in different human body fluids have been widely studied. Yet, much fewer studies focus on small RNA.

MicroRNAs (miRNAs) are short single-stranded small RNAs that regulate target mRNA expression by cleavage or translational inhibition. It commonly exists in all types of cells. Age and disease-related up-/down-regulation of the miRNA accumulation in adult neurons can lead to changes in their survival, functions, and communication (Konovalova et al., 2019). The fact that the exosome can carry specific bio-signatures linked to disease pathogenesis has sparked great interest in potential usage in diagnostics and therapeutics. However, due to the nature of sRNA diversity in exosomes, exosomal sRNA patterns are easily interfered, which lacks reproducibility between experiments (Witwer et al., 2013; Enderle et al., 2015). Next-generation sequencing is therefore introduced to comprehensively describe characters of isolated exosome and to eliminate bias (Gonzales et al., 2009; Taylor et al., 2011; Rekker et al., 2014).

Several techniques have been invented to isolate exosome from different cell types. Traditional centrifugation-based approaches are most commonly used techniques and considered as the gold standard for general exosome isolation (Thery et al., 2006). However, they require large sample sizes, appropriate equipment and are time-consuming. Therefore, an alternative method with better convenience and reproducibility is urgently needed. Precipitation and column-based exosome isolation methods are optional strategies that are relatively time saving, cost effective, and efficient.

In the present study, we aimed to explore expression levels of plasma exosomal miRNAs, and to detect new biomarkers for AD and PD in plasma exosome. Besides, we evaluated the miRNA profile for reproductivity of the exosome isolating experiments by using different exosome isolating methods. We reported differential expression level of plasma exo-miRNA in neurodegenerative diseases and the potential biomarkers for AD and PD.

## Materials and Methods

### Sample Collection and Ethics Statement

In this study, all peripheral blood samples were collected from 34 normal controls, 5 AD donors and 7 PD donors (46 in total). Supplementary Table S1 provides the age, gender, and diagnosis of these participants. The diagnosis of AD cases was based on the criteria of the National Institute on Aging and Alzheimer’s Association, and the diagnosis of PD was based on the United Kingdom Brain Bank criteria with some clinical supportive examinations (i.e., dyskinesia, rest tremor).

All participants were under approval from Affiliated Hospital of Jining Medical University IRB (2017-KE-B001) and BGI IRB (No. BGI-IRB16019), and each of them was properly consented before sample collection. All samples had been anonymized for research purposes. The control samples HBRR (Human Brain Reference RNA, Cat. No. AM6050) were supplied by Thermo Fisher Scientific.

### Collection of Neuronal-Derived Exosomes From Blood

Samples were collected following the fasting blood standard protocol^1^. Exosome isolation was carried out using two commercially available kits: exoRNeasy Serum/Plasma Maxi Kit (QIAGEN, Hilden, Germany) (SC); ExoQuick Plasma prep and Exosome isolation kit (SBI, Palo Alto, United States) (EQ). For each sample, we used 1 ml plasma for one reaction of SC kit and 0.5 ml plasma for one reaction of EQ kit.

For employing EQ method, 5 μl of Thrombin 500U/ml (SBI, Palo Alto, United States) was added into 0.5 ml prefiltered plasma. The mixture was incubated for 5 min and centrifuged for 5 min at 8,000 × g. We added 1/4 volume of ExoQuick Solution to the supernatant and incubated it at 4°C for 30 min. The mixture was then centrifuged at 1,500 × g for 30 min. Finally, the pellets were re-suspended with nuclease-free PBS.

For employing SC method, we prefiltered plasma and mixed the flow-through with 2 × binding buffer. Then the solution was added to the exoEasy membrane affinity column and centrifuged for 1 min at 500 × g. The pellets were washed with washing buffer by centrifuging and discarding the flow-through. The pellets were isolated exosome and re-suspended with nuclease-free PBS.

### Small RNA Extraction, Library Preparation and Small RNA Sequencing

The small RNA was extracted according to the manufacturer’s instructions (QIAGEN, Hilden, Germany, Cat No. 217084). The RNA yield was measured by NanoDrop 2000 Spectrophotometer (Thermo Fisher Scientific). Small RNA sequencing libraries were constructed using the MGIEasy Small RNA Library Prep Kit (MGI, Shenzhen, China). Total of 1 G reads were generated for each sample consequently. The data reported in this study are available in the NCBI BioProject (Accession Number: PRJNA587017) and CNGB Nucleotide Sequence Archive (Accession Number: CNP0000728).

### Small RNA Data Analysis

**FIGURE 1:**
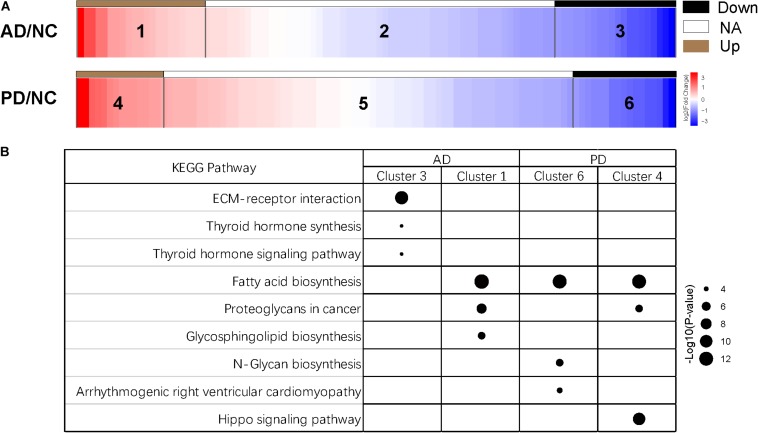
Clusters of miRNA expression and enriched pathways. **(A)** Heatmap of differentially expressed miRNAs of six clusters. Down, miRNAs with expression of log_2_(AD/NC) or log_2_(PD/NC) < -1; NA, miRNAs with expression of -1 < log_2_(AD/NC) or log_2_(PD/NC) < 1; Up, miRNAs with expression of log_2_(AD/NC) or log_2_(PD/NC) > 1. **(B)** KEGG pathway of miRNAs from four clusters. Target genes of miRNAs were predicted by miRanda, TargetScan, and RNAhybrid individually. Joint target genes were selected and KEGG pathway enrichment analysis was carried out. Top three pathways of each cluster were shown. The size of the dots represents the -log_10_(*p*-value) of each pathway.

After removing adaptor sequences and filtering out low-quality reads, the cleaned sRNAs reads were mapped against human reference genome hg19 UCSC and Rfam (version 11.0)^2^ database. The remaining reads were aligned and annotated according to precursor and mature miRNAs listed in miRBase (Altschul et al., 1990). To tabulate miRNA expression levels, read counts were tabulated from the expression tabulation purpose mapped reads. The size factor used to normalize the read counts was calculated with the DESeq2 package, and the normalized read counts were then analyzed by the DESeq2 to identify differentially expressed miRNAs (Love et al., 2014). Pairwise library comparisons for each miRNA were performed and fold change of each miRNA was generated. Then, top 100 abundant miRNAs were selected, and six groups were clustered according to miRNA expression in AD and PD samples (Figure 1). Cluster 1 and 4 represents the fold change ≥ 2; Cluster 3 and 6 represents the fold change ≤−2; Cluster 2 and 5 represents -2 ≤ the fold change ≤ 2. Next, miRNAs that had a *p* ≥<0.001 and an absolute log fold-change ≥ indicated a differential expression between the two conditions being compared.

### Target Prediction and KEGG Pathway Analysis

The targets of miRNAs were predicted separately by miRanda, TargetScan, and RNAhybrid (Kruger and Rehmsmeier, 2006; Betel et al., 2010; Agarwal et al., 2015). From miRanda, TargetScan, and RNAhybrid, 23253, 9024, and 24906 targets were predicted from all 1108 identified miRNAs, respectively. 8913 target genes were found in all three software. KEGG pathway enrichment (*p-*value) was calculated using overlapped genes from 100 top abundant miRNA by DIANA-miRPath (Vlachos et al., 2015).

### Statistical Analysis

The pairwise correlation coefficient and corresponding figures were calculated and plotted by R package: corrgram. Co-inertia analysis (CIA) was performed by cia function from R package: made4. Monte-Carlo test was performed to determine the significance of relationship. Correlative analysis was performed using a linear regression model. All tests were two-tailed, and the significant difference was set at *p* < 0.05.

## Results

### The miRNA Expression in AD and PD Samples

We collected human plasma from 5 AD patients, 7 PD patients and 20 normal (NC) participants. The age of each participants and Mini-Mental State Examination (MMSE) of AD patients were presented in Supplementary Table S1. After isolating exosome from plasma samples by using ExoQuick^TM^ (EQ) kit, small RNA extraction was performed and next-generation sequencing was carried out in BGISEQ-500 platform (Mak et al., 2017). In total, 1 G on average raw data per sample was generated. After the removal of rRNA, the data was mapped to miRbase for human miRNA annotation. Counts of each miRNA were calculated. Large individual variances could be observed in the heatmap of the miRNA expression suggesting that sample size could be a limit in this study (Supplementary Figure S1).

After selecting miRNAs of top 100 abundance, six clusters were generated according to the different signal intensity of exosomal miRNAs in AD and PD compared to NC (Figure 1). We considered miRNAs with log_2_(fold change) > 1 or < −1 as differentially expressed ones. More than half miRNAs were in cluster 2 and 5 (57 miRNAs in cluster 2, 68 miRNAs in cluster 5). We found 22 and 14 miRNAs (cluster 1 and 4) that were up-regulated while 21 and 18 miRNAs (cluster 3 and 6) were down-regulated in AD and PD samples, respectively (Figure 1A and Supplementary Table S2). Notably, only 7 miRNAs were altered consistently in AD and PD samples (Supplementary Table S3). Compared to NC samples, the expression levels of *miR-27a-3p* and *miR-584-5p* were elevated in both AD and PD samples, while those of five miRNAs (*miR-942-5p*, *miR-92b-3p, miR-375, miR-122-5p*, and *miR-1468-5p*) were declined in both AD and PD samples (Supplementary Figure S2). It suggested that though many miRNAs with different expression levels were found in AD/NC and PD/NC, there were still some differences of exo-miRNA performance between two neurodegenerative diseases.

Subsequently, we used miRanda, TargetScan, and RNAhybrid to predict targets of miRNAs, and 8913 targets were predicted from all three methods (Supplementary Figure S3). Then, we selected targets of the top 100 abundant miRNAs, and used DIANA-miRPath (Version 3.0) tool to calculate *p*-value of the KEGG pathway enrichment of cluster 1, 3, 4, and 6 (Kanehisa and Goto, 2000; Kruger and Rehmsmeier, 2006; Betel et al., 2010; Agarwal et al., 2015; Vlachos et al., 2015). The information of pathways was presented in Supplementary Table S4. Three pathways with the highest *p*-value were selected from each cluster (Figure 1B). Interestingly, miRNAs in cluster 3 were enriched in the extracellular matrix (ECM)-receptor interaction indicated that these suppressed miRNAs might involve in a direct or indirect control of cellular activities such as adhesion, migration, differentiation, proliferation, and apoptosis (Hara et al., 2017). In other three clusters, fatty acid biosynthesis was the most abundant pathway (Figure 1B). Fatty acid biosynthesis was closely related with mitochondria in which energy production is strongly disturbed in neurons of AD and PD patients. The altered fatty acid related miRNA expression in exosome could be a widespread response to certain diseases. Additionally, miRNAs in cluster 4 were enriched in hippo signaling pathway which has not been reported to be relevant with Parkinson’s disease (Figure 1B).

### Down-Regulation of Exo-miRNA in AD and PD Samples

**FIGURE 2:**
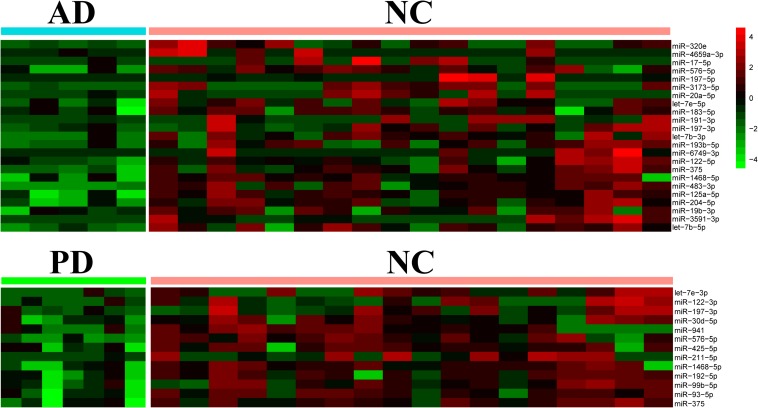
Heatmap of differential expression levels of miRNAs in AD and PD patients. The color scale shown on the right illustrates the expression level (log_2_) of a miRNA across all samples. Each row represents significantly declined miRNAs (*p* < 0.05) in AD or PD patients including 23 declined miRNAs in AD patients and 13 declined miRNAs in PD patients.

Differential expression of miRNAs was calculated from all identified miRNAs by using DESeq2, *p* < 0.05 (Supplementary Table S3; Love et al., 2014). More than half of differentially expressed miRNAs were declined in both AD and PD samples. Out of 23 miRNAs down-regulated in AD samples, 4 miRNAs were down-regulated with PD as well while 1 miRNA was up-regulated in PD patients (Table 1). Heat map presented a variety of down-regulation of exo-miRNA expression in AD and PD patients including 18 miRNAs from AD patients and 9 miRNAs from PD, as well as 4 commonly down-regulated miRNAs (Figure 2). It was consistent with previous studies which indicated that this could be a common phenomenon of neurodegenerative disease patients, as reflected in exosomal miRNA expression levels (Keller et al., 2011; Hewel et al., 2019; Sadlon et al., 2019).

**TABLE 1:** Differential expressed exo-miRNA in AD and PD samples.

AD	log2FC(AD/NC)	*P*-value (AD)	PD	log2FC(PD/NC)	*P*-value (PD)
miR-197-3p	–4.76	0.000	miR-197-3p	–3.11	0.008
miR-576-5p	2.75	0.024	miR-576-5p	–3.37	0.001
miR-1468-5p	–3.04	0.003	miR-1468-5p	–2.82	0.001
miR-375	–2.01	0.004	miR-375	–1.66	0.007
let-7e-5p	–2.10	0.003	let-7e-5p	2.83	0.000
miR-483-3p	–7.07	0.000	miR-211-5p	–3.89	0.002
miR-3173-5p	–6.38	0.000	let-7e-3p	–3.44	0.003
miR-320e	–5.66	0.000	miR-122-3p	–2.90	0.031
miR-197-5p	–5.52	0.004	miR-941	–2.67	0.013
miR-193b-5p	–4.92	0.000	miR-30d-5p	–2.46	0.002
miR-6749-3p	–4.91	0.014	miR-192-5p	–1.85	0.002
miR-20a-5p	–4.77	0.009	miR-93-5p	–1.84	0.001
miR-191-3p	–4.63	0.013	miR-425-5p	–1.58	0.011
miR-4659a-3p	–4.54	0.018	miR-99b-5p	–1.26	0.028
let-7b-3p	–4.09	0.002	let-7i-5p	0.92	0.023
miR-17-5p	–3.99	0.042	miR-652-3p	0.97	0.022
miR-3591-3p	–3.97	0.011	miR-4732-3p	1.73	0.020
miR-125a-5p	–3.38	0.000	miR-6131	2.33	0.013
miR-204-5p	–2.79	0.000	miR-3184-3p	2.86	0.014
miR-122-5p	–2.37	0.007	miR-378g	3.64	0.001
miR-19b-3p	–2.16	0.040			
miR-183-5p	–1.64	0.036			
let-7b-5p	–1.37	0.018			
miR-22-3p	0.98	0.015			
miR-151a-5p	1.06	0.045			
miR-27b-3p	1.19	0.030			
miR-21-5p	1.52	0.032			
miR-27a-3p	1.62	0.031			
miR-146a-5p	1.85	0.017			
miR-28-3p	2.30	0.038			
miR-379-5p	2.38	0.032			
miR-23a-3p	2.55	0.000			
miR-199a-3p	3.15	0.010			
miR-369-5p	3.16	0.001			
miR-382-5p	3.72	0.033			
miR-378i	4.31	0.008			
miR-423-5p	4.35	0.003			

**Underlined miRNAs differentially expressed in both AD and PD samples.**

### Both Reported and Novel AD-/PD-Biomarkers Were Found in Exo-miRNA Profiles

**FIGURE 3:**
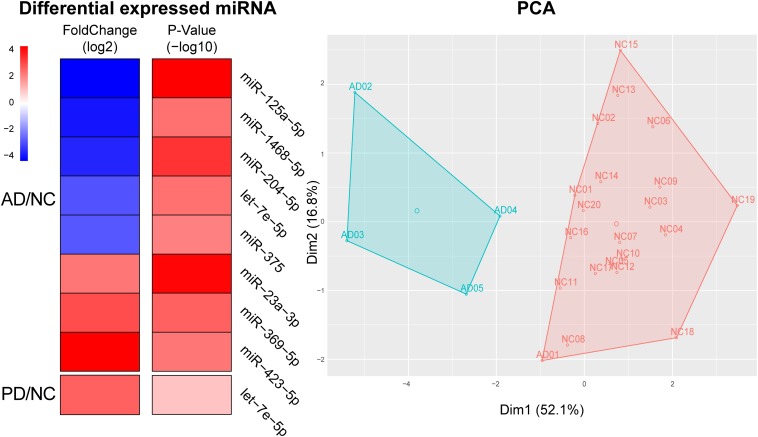
Using significantly altered expression of miRNAs to distinguish AD from NC. Left, Heatmap of significant altered levels of miRNA expression in AD and PD samples. DESeq2 was used to identify significantly up-/down-expressed levels of miRNAs. Fold change was presented by log_2_ and *p*-value was presented by log_10_. PCA and k-means based clustering was performed to distinguish AD samples from NC samples by using significantly expressed levels of miRNAs.

From the differential expressed miRNAs in Table 1, we selected candidates in the top 100 abundance miRNAs. Eight miRNAs were finally found differentially expressed between AD and NC in which three miRNAs, *miR-423-5p* (*p* = 2.86 × 10^–3^), *miR-369-5p* (*p* = 1.46 × 10^–3^), *miR-23a-3p* (*p* = 6.17 × 10^–5^) were significantly elevated in AD samples compared to NC samples and five miRNAs *miR-204-5p* (*p* = 2.68 × 10^–4^), *miR-125a-5p* (*p* = 5.09 × 10^–5^), *miR-1468-5p* (*p* = 2.63 × 10^–3^), *miR-375* (*p* = 4.42 × 10^–3^), *let-7e-5p* (*p* = 2.57 × 10^–5^) were significantly declined in AD samples (Figure 3). Only one miRNA, *let-7e-5p* (*p* = 6.25 × 10^–5^) was differentially expressed between PD and NC (Figure 3). PCA was carried out and 4 out of 5 AD samples can be distinguished from NC samples, suggesting that using these plasma sourced exosomal miRNAs as biomarkers may distinguish AD samples from control ones.

Within eight significantly different miRNAs, *let-7e-5p*, *miR-125a-5p*, *miR-23a-3p*, and *miR-375* were reported as AD biomarkers in previous independent studies: *let-7e-5p*, *miR-125a-5p*, *miR-23a-3p*, and *miR-375* were marked in whole blood, plasma and plasma exosome, serum and plasma exosome, and serum, respectively (Burgos et al., 2014; Dong et al., 2015; Satoh et al., 2015; Gao et al., 2017). Four miRNAs including *miR-1468-5p*, *miR-204-5p*, *miR-369-5p*, and *miR-423-5p*, were newly found differential in AD. This exo-miRNA performance could therefore be used for the diagnosis of the pathological changes in AD.

### Validation of Candidate miRNAs by Another Exosomal Isolation Kit

To evaluate how the exosome isolation method influences small RNA performance, we tested another commercially available exosome isolation kit: column-based kit (exoEasy spin column, SC). Additionally, small RNA in plasma without exosome isolation was also extracted (called PC). Small RNAs yield of samples from three approaches was measured by NanoDrop 2000 Spectrophotometer (Thermo Fisher Scientific). Library preparation and next-generation sequencing were followed by using BGI-seq 500 platform (Table 2). Small RNA data were generated from exosome isolation by using precipitation-based method, and column-based method were named as EQ, SC, while data generated in plasma without exosome isolation was named PC.

**TABLE 2:** Exosome isolation workflow.

	SC	EQ	Plasma
Exosome isolation	1. Filtered plasma 1 ml. 2. ExoEasy spin column. 3. Elute and collect the EVs.	1. Filtered plasma 0.5 ml. 2. Thrombin for fibrin treatment. 3. ExoQuick^TM^ solution for precipitation. 4. Resuspend the EVs pellet.	N/A
miRNA extraction	1. Small RNA extraction following the QIAzol protocol. 2. Small RNA measurement.		
Library preparation and sequencing	1. 200 ng of total RNA, MGIEasy Small RNA Library Prep Kit. 2. BGISEQ-500 platform. 3. Acquire > 25M reads per each sample.		

**FIGURE 4:**
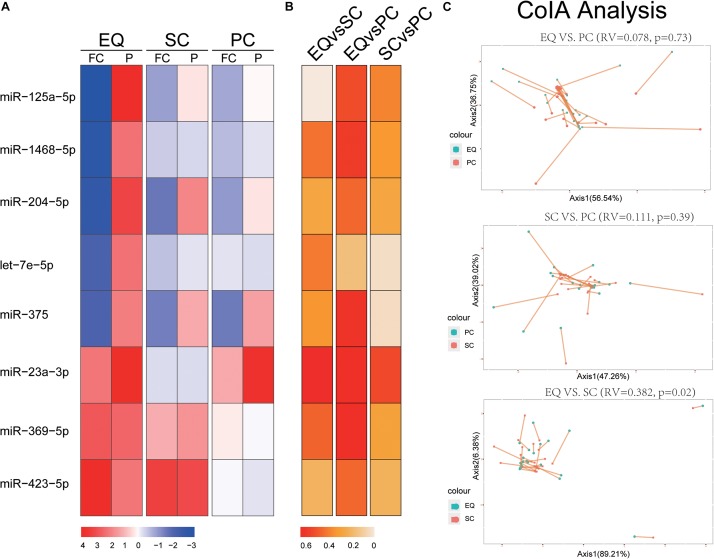
Validation of candidate miRNAs by SC and PC. **(A)** Fold change (FC) and *p*-value of candidate miRNA expression in AD samples, FC and *p*-value were calculated by DESeq2. **(B)** Spearman Correlation coefficients of miRNA expression between EQ, SC, and PC. **(C)** Co-inertia analysis of the relationships between EQ, SC, and PC. Monte-Carlo test was carried out, and the significance of correlation could be observed between EQ and SC (*p* < 0.05). Each sample is represented with two dots connected by a thin line.

We extracted the expression of 8 miRNA that were found significantly altered in EQ data from SC and PC data (Figure 4). The fold changes of five down-regulated miRNAs (*miR-125a-5p, miR-1468-5p, miR-204-5p, let-7e-5p, miR-375*) were consistent between EQ, SC and PC. The fold changes of *miR-369-5p* and *miR-423-5p* were consistent between EQ and SC (Figure 4A). Correlation analysis showed *miR-1468-5p* and *miR-23a-3p* had higher correlation coefficients than other miRNAs (Figures 4B). Co-inertia analysis of the relationships between EQ, SC, and PC was carried out by using the expression of 5 AD and 20 NC samples (Figure 4C). Significant correlation was detected between EQ and SC (*p* = 0.02), indicating the expression of candidate miRNAs can be validated by exo-miRNA profile from another isolating method (Figure 4C).

### The Reproductivity of Exo-miRNA Profile

We further evaluated the isolation methods by their RNA yield, sequence data quality and miRNA expression. Additional 14 control samples were added into this study. In total, 46 human plasma samples were collected including 5 AD patients, 7 PD patients, and 34 NC participants.

Human Brain Reference RNA (HBRR) samples were added into sRNA libraries as external markers in order to eliminate the bias of each sequencing lane. It was observed that over 20 million reads per sample were mapped to human genome, and most of the reads enriched in the range of 18–25 nt (Supplementary Figures S4A,B). Considering the fact that miRNA expression of all HBRR samples were highly correlated, it could be concluded that the batch effect was negligible, and the quality control of the library preparation and sequencing platform was eligible for our study (Supplementary Figures S4C–E).

**FIGURE 5:**
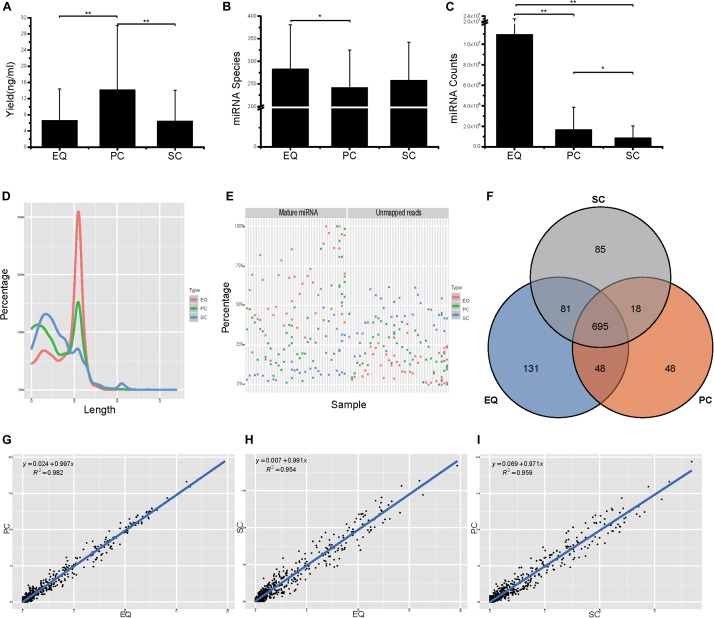
Analysis of the differences in exosomal miRNA profiles among three isolation methods. **(A)** Small RNA yield efficiency by three methods. **(B)** Identified miRNA species by three methods, average of miRNA species from 46 samples. **(C)** MiRNA total counts by three methods, average of total miRNA counts from 46 samples. **(D)** Average read length distribution by three methods. **(E)** Aligned miRNA reads and unmapped reads of 46 samples by three methods. **(F)** Venn diagram presents all identified miRNAs that are common or unique in the three method datasets. Most (695) miRNAs were common to the three sets. **(G–I)**, Scatter plots reveal correlations between EQ and PC **(D)**, EQ and SC **(E)**, SC and PC **(F)**. Each scatter plot represents relative expression of miRNA profiles obtained by two methods. * represents *p* < 0.05 in Student’s *T-*test. ** represents *p* < 0.01 in Student’s *T*-test.

Then, we compared the three datasets, i.e., EQ, SC, and PC, from 46 samples from small RNA yield, identified miRNA number and miRNA counts. The total yields of 138 (46 × 3) samples from EQ, SC, and PC were measured and the average yield of PC was significantly larger than those of EQ and SC (*p* < 0.01) while no difference had been found between EQ and SC (Figure 5A). More than 250 miRNAs were identified in EQ samples aligned to miRbase^3^, which was notably larger than that in PC samples (*p* < 0.05). No significant differences could be found either between EQ and SC or between PC and SC (Figure 5B). Total number of aligned miRNA counts displayed an order of EQ > PC (*p* < 0.01) > SC group (*p* < 0.05) (Figure 5C). Therefore, EQ data presented remarkably larger data size while no fundamental difference of sRNA yield was found between EQ and SC methods.

The EQ reads enriched in 18–25 nt while a great number of SC and PC reads showed enrichment in the <18 nt range (Figure 5D). Moreover, EQ data had the highest percentages of mature miRNA reads and the lowest percentages of unmapped reads, suggesting that EQ group had better data quality compared to SC and PC (Figure 5E). Among all detected miRNAs, 695 common miRNAs were found in all three data while 85, 131, 48 unique miRNAs were observed in SC, EQ, and PC samples, respectively (Figure 5F and Supplementary Table S3). At last, the pairwise correlation coefficient was calculated among the three pairs of samples. The correlation (*R*^2^) values were 0.982 between EQ and PC groups, 0.954 between EQ and SC groups and 0.959 between SC and PC groups (Figures 5G–I). Overall, these verified the reproductivity of exo-miRNA profile and better data quality of EQ data.

### MiRNA Expression Can Distinguish SC From EQ and PC

**FIGURE 6:**
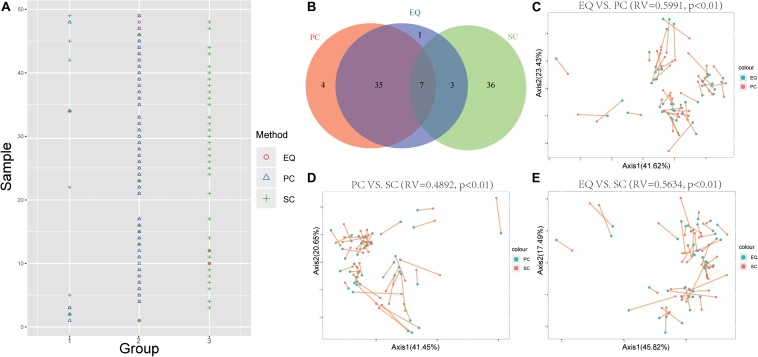
Correlation of EQ, PC, and SC profiles. **(A)** Grouping information for each sample by PCA. Colors and shapes represent three exosome isolation methods. Y-axis shows the sample number. X-axis shows the grouping information. **(B)** Venn diagram represents EQ, PC, and SC samples that are grouped into the same clusters. **(C)** Co-inertia analysis (CIA) of relationships between the EQ and PC miRNA expressional PCA. **(D)** CIA of relationships between the PC and SC miRNA expressional PCA. **(E)** CIA of relationships between the EQ and SC miRNA expressional PCA. **(C–E)** *p*-value < 0.01 from 99 Monte-Carlo simulations.

To further study whether EQ, SC, PC samples could be distinguished by the miRNA expression profiles, the k-means clustering was carried out to classify data generated from each sample by EQ, PC and SC methods. Unsurprisingly, 42 out of 46 EQ and PC samples can be clustered into group 2 (Figures 6A,B). Additionally, co-inertia analysis between the miRNA profiles of EQ, PC and SC samples revealed a significant co-variation (Figures 6C–E; Zhao et al., 2018). Significant variances were observed in all three comparisons (*p* < 0.01) and the RV coefficients were 0.5991, 0.4892, and 0.5634 between EQ and PC, between PC and SC, and between EQ and SC, respectively. Rather than SC samples, the miRNA profiles of EQ samples were more related to those of PC samples (Figures 6C–E).

**FIGURE 7:**
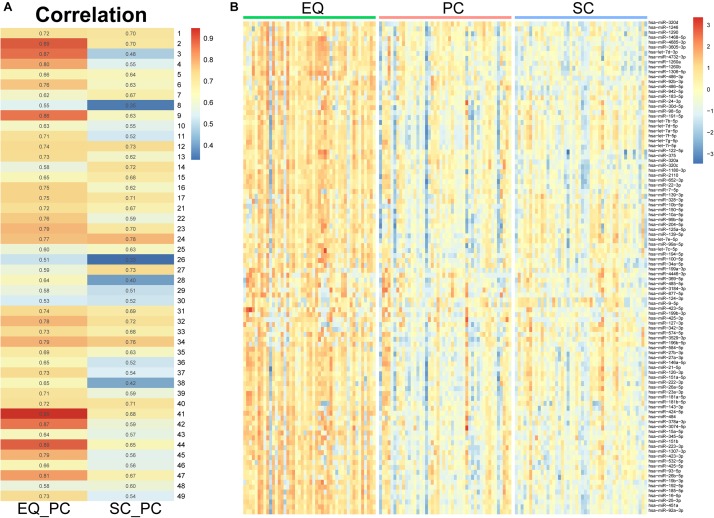
miRNA expression under three isolation methods. **(A)** Spearman correlation coefficient was calculated from miRNA expression data in the pair of EQ-PC and SC-PC in each sample. EQ data exhibited higher correlation with PC data than SC with PC. **(B)** Heatmap of miRNA expression in 46 samples. The top 100 abundant miRNAs were displayed. Higher expression levels were obviously observed in EQ data.

Since the three datasets were generated from one sample, we analyzed correlation between miRNA expression levels of EQ, SC, and PC (Figure 7A). In 46 samples, the spearman correlation coefficients of miRNA expression between EQ and PC were from 0.51 to 0.95, while those between SC and PC were from 0.33 to 0.78. Consistent with the results above, the overall correlation coefficients between EQ and PC were higher than those between SC and PC. The corresponding miRNA expression levels of EQ data were also significantly higher than those of SC data (Figure 7B). Then, we could conclude that better exo-miRNA data were generated by using the EQ isolating method.

## Discussion

Neurodegenerative diseases such as AD and PD originate from a loss of neurons in the CNS (Tan et al., 2015). Using new methods to assist diagnosis of AD and PD is still under development. In the present study, we compared miRNA profiles of 5 AD and 7 PD samples to those of control samples. Eight miRNAs were suggested as biomarkers of neurodegenerative diseases. However, the sample size of AD and PD limits the accuracy of the biomarker selection. We downloaded small RNA sequencing data including 48 AD and 29 PD samples from two previous studies (Leidinger et al., 2013; Hoss et al., 2016). The AD samples were collected from the whole blood, and the PD samples were collected from the prefrontal cortex. Considering 100 most abundant miRNAs, 90 miRNAs could be identified from the download data of 48 AD and 22 control samples while 94 miRNAs could be identified from the data of 29 PD and 33 control samples (Supplementary Table S2). In 48 AD samples, 6 down-regulated and 1 up-regulated miRNAs were identified which is consistent with our results: much more down-regulated miRNAs can be identified in AD samples. However, the expression of significantly altered miRNA in our data didn’t display consistency with download data (Supplementary Table S2). It could be caused by the difference of sample types indicating that the expression of exo-miRNA presents different pattern compared to those from whole blood and the brain.

To obtain the more robust biomarkers, multiple methods of differential expression analysis were applied by using EdgeR and Limma R packages (Robinson et al., 2010; Ritchie et al., 2015). Then, 15 miRNAs and 3 miRNAs were identified in AD and PD samples by all three software (Supplementary Figure S5 and Supplementary Table S5). Results from EdgeR and DESeq2 shared more common miRNAs in both AD and PD samples. Less than half of the miRNAs from Limma can be found by EdgeR or DESeq2 suggesting that too many false positive results were produced by Limma. Combining with the fact that the original design of Limma was to analyze Chip-seq data, Limma did not fit this data. In AD samples, 9 and 24 unique miRNAs were identified by DESeq2 and EdgeR, while 28 miRNAs were commonly found by DESeq2 and EdgeR (Supplementary Figure S5). Nearly three quarters of DESeq2 identified miRNAs could be mutually found by EdgeR, and results from DESeq2 were trustworthy.

Exosomes do not carry the necessary molecules to accomplish cell-independent miRNA biogenesis as previously reported, suggesting that the release of exo-miRNAs aims to regulate gene expression in target cells or extracellular milieu, rather than targets inside exosomes (Melo et al., 2014; Jeppesen et al., 2019). In this study, we found down-regulated miRNAs enriched in different AD and PD clusters (Figure 1B). Targets of seventeen miRNAs from cluster 3 were enriched in ECM-receptor interaction signaling pathway (Supplementary Table S4). It indicated dramatic alteration in post-transcriptional regulation of extracellular environment. Fatty acid biosynthesis pathway involved six miRNAs from cluster 1, three miRNAs from cluster 4, and three miRNAs from cluster 6 (Supplementary Table S4). Therefore, these enriched pathways may exhibit that the miRNA-level response mainly focused on extracellular environment and fatty acid biosynthesis (Figure 1B).

Expression level of *miR-424-5p* elevated in AD and declined in PD (Supplementary Table S2). Fatty acid synthase (FASN) and Acyl-CoA synthetase long chain family member 3/4 (ACSL3/4), which are target genes of *miR-424-5p*, are strongly involved in energy metabolism in brain (Mashek et al., 2007; Long et al., 2013). It could be a new biomarker for neurodegenerative diseases. Overexpression of *miR-423-5p* significantly increased in distant brain metastasis (Sun et al., 2018). It is a biomarker of brain metastasis and a therapeutic target in lung adenocarcinoma (Sun et al., 2018). Up-regulation of *miR-423-5p* in AD samples could be a response to brain dysfunction, it also has potential to be a novel plasma marker for AD.

Additionally, we need biomarkers to distinguish neurodegenerative diseases from each other. Exosomal protein and miRNA isolated from CSF sometimes lead to contrary results between AD and PD (Kim et al., 2016; Jia et al., 2019). In our data, *let-7e-5p* was found elevated in PD samples and declined in AD samples, indicating that *let-7e-5p* could be a biomarker to differentiate the sub-type of neurodegenerative disease. Neurotoxic miRNA family *let-7* has long been discovered related to initiation innate immune pathways and apoptosis in the CNS (Lehmann et al., 2012; Derkow et al., 2018). Extracellular *let-7b* activates the RNA-sensing Toll-like receptor (TLR) 7 and induces neurodegeneration (Lehmann et al., 2012). As the miRNAs with redundant functions could be cooperative to buffer transcriptomic balance, expression level of *let-7e* might be suppressed by high level of *let-7b* in AD (Fischer et al., 2015). PD patients also suffer from neurodegeneration, expression of *let-7* should elevate as it is in AD patients, yet no evidence indicates higher level of *let-7b* in PD samples (Dawson and Dawson, 2003). That could be a reason why expression level of cooperative *let-7e* was lower in AD and higher in PD.

We compared exo-miRNA expression levels between AD and PD samples (Supplementary Table S6). Eight miRNAs were differentially expressed between AD and PD. Six miRNAs elevated in PD, and two miRNAs elevated in AD. Among 8 significantly altered miRNAs, three were *miR-548* members. The *miR-548* (*ap, ad-5p, k*) is an anti-oncogenic regulator, and miR-548 can induce cell apoptosis in the breast cancer cell (Ke et al., 2016). Three *miR-548* (*ap-3p, ad-5p, k*) elevated in PD, suggesting that PD patients might have greater oncogenic stress than AD patients.

Traditional exosome isolation methods using ultracentrifuge do not satisfy the clinical need for high-through and rapidity (Tang et al., 2017). Commercially available kits would overcome the obstacles between related research and clinical application, as well as the cost of diagnosis. We tested two commercially available kits and compared them with small RNA profile in plasma. Based on the sequence data, miRNA in exosome exhibit higher expression levels and better quality than that in plasma. It suggests that the biomarkers in exosome should have better diagnostic efficiencies than in plasma. Besides, we tested whether the storage would affect exosomal sRNA profile. Four plasma samples were stored at -80°C for 1, 2, and 3 months followed by sRNA sequencing. Except EQ samples, miRNA counts of PC and SC samples didn’t show a very clear pattern with time (Supplementary Figure S6). In spite of this, plasma samples should be stored at −80°C no more than 2 months in order to get good miRNA quality.

Finding biomarkers in blood exosome is a quite popular and practical direction of neurodegenerative diseases early diagnosis. In this study, we sequenced exo-miRNA from human blood of AD and PD patients. The reproducibility of small RNA results has always been a barrier of identifying stable biomarkers. We tested the plasma exo-miRNA expression by applying multiple small RNA extraction methods. Three datasets, EQ, SC, and PC, confirmed the stability of the pipeline used in this study suggesting that our results represented the exo-miRNA profiles in AD and PD patients. However, due to the limitation of sample size and the variety of miRNA expression, the biomarkers we proposed need further verification in longitudinal studies.

## Data Availability Statement

The datasets generated for this study can be found in the NCBI BioProject (Accession Number: PRJNA587017) and CNGB Nucleotide Sequence Archive (Accession Number: CNP0000728).

## Ethics Statement

The studies involving human participants were reviewed and approved by Affiliated Hospital of Jining Medical University IRB (2017-KE-B001) BGI IRB (No. BGI-IRB16019). The patients/participants provided their written informed consent to participate in this study.

## Author Contributions

All authors listed have made a substantial, direct and intellectual contribution to the work, and approved it for publication.

## Conflict of Interest

The authors declare that the research was conducted in the absence of any commercial or financial relationships that could be construed as a potential conflict of interest.

## Footnotes

1 https://medlineplus.gov/lab-tests/fasting-for-a-blood-test/

2 http://rfam.xfam.org/

3 http://www.mirbase.org/

## Supplementary Material

FIGURE S1 Heatmap of the miRNA expression in AD, PD, and NC.

FIGURE S2 Seven miRNAs consistently altered in both AD and PD.

FIGURE S3 Target prediction results by three software.

FIGURE S4 HBRR samples showed that batch effect was negligible.

FIGURE S5 Identification of differential miRNA expression.

FIGURE S6 Small RNA data quality of samples under -80°C storage.

TABLE S1 Characteristics of participants.

TABLE S2 Differential expressed miRNAs in AD and PD identified by DEseq2.

TABLE S3 Full list of miRNA expression.

TABLE S4 KEGG results of each cluster.

TABLE S5 List of differentially expressed miRNA generated by three software.

TABLE S6 Expression of 8 miRNAs in AD and PD samples.
