# Supplementary figures and images for: Differential Expression of Plasma Exo-miRNA in Neurodegenerative Diseases by Next-Generation Sequencing
Source: Front Neurosci. 2020 May 7;14:438. doi: 10.3389/fnins.2020.00438 (PMC7227778; doi:10.3389/fnins.2020.00438)

AD

NC

PD

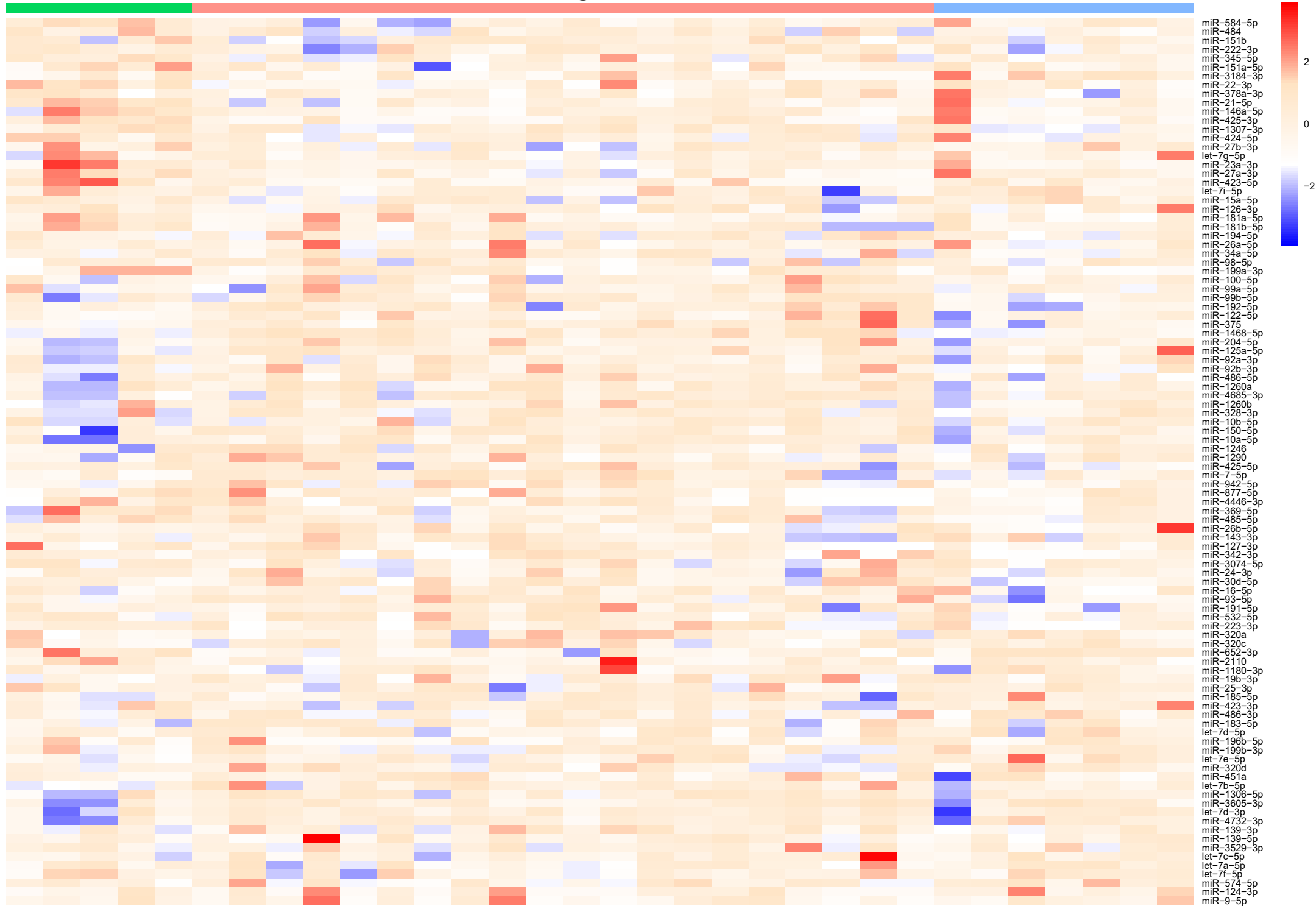

Supplement: FIGURE S1 — Heatmap of the miRNA expression in AD, PD, and NC. [file Data_Sheet_1.PDF]

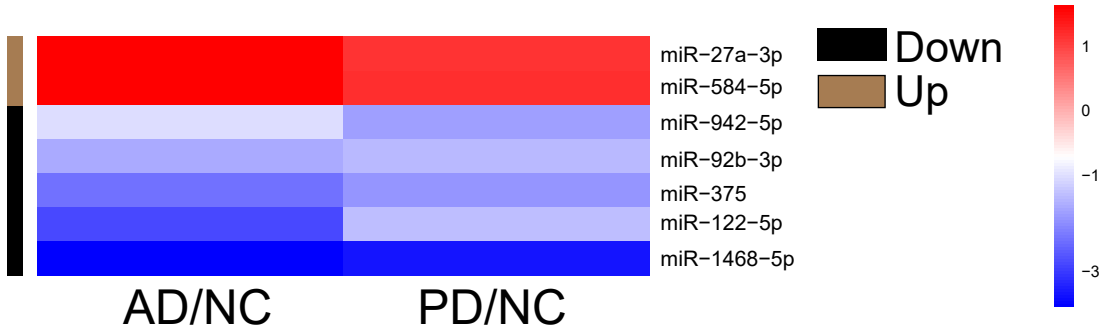

Supplement: FIGURE S2 — Seven miRNAs consistently altered in both AD and PD. [file Data_Sheet_2.PDF]

RNAhybrid

TargetScan

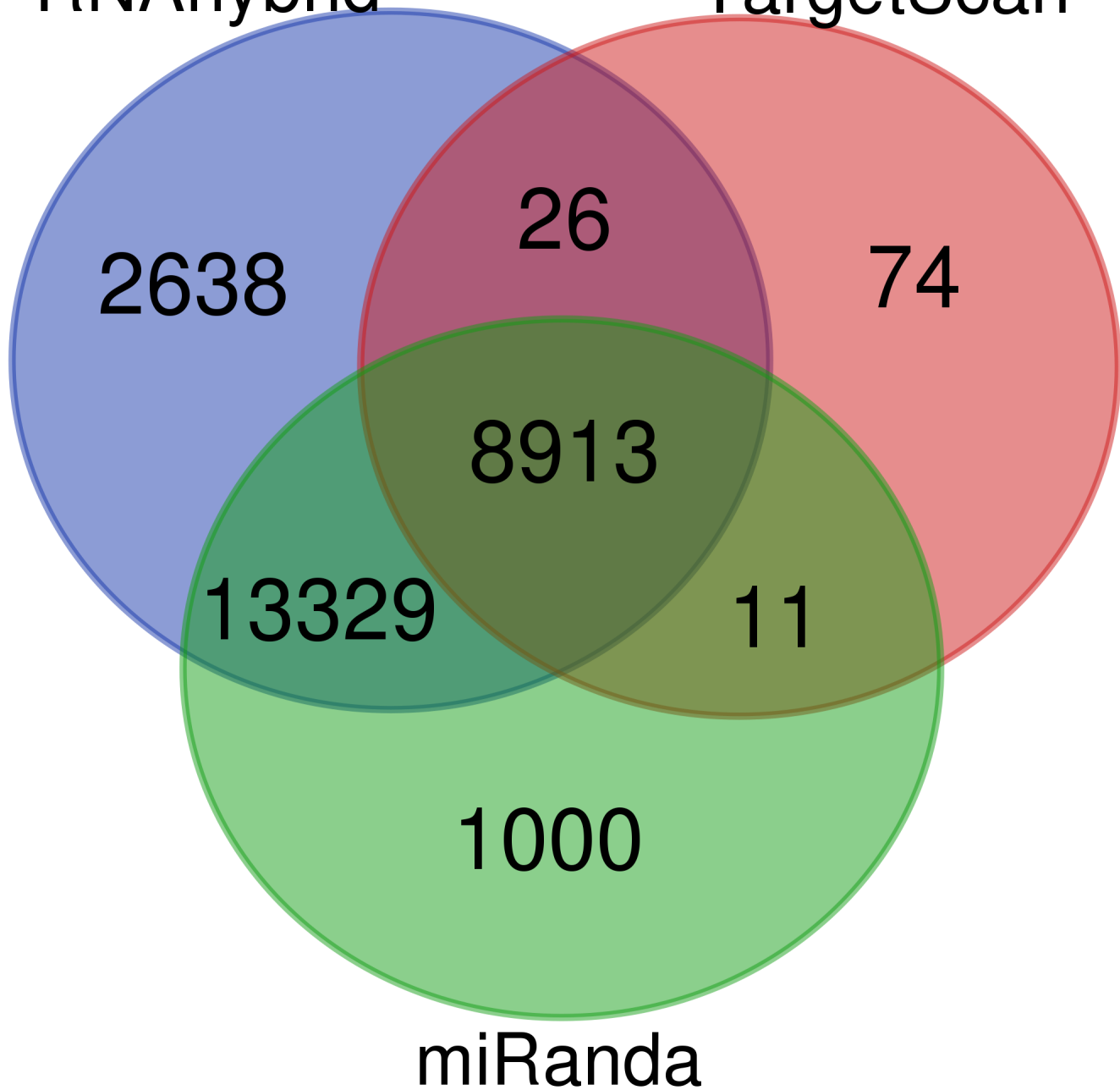

Supplement: FIGURE S3 — Target prediction results by three software. [file Data_Sheet_3.PDF]

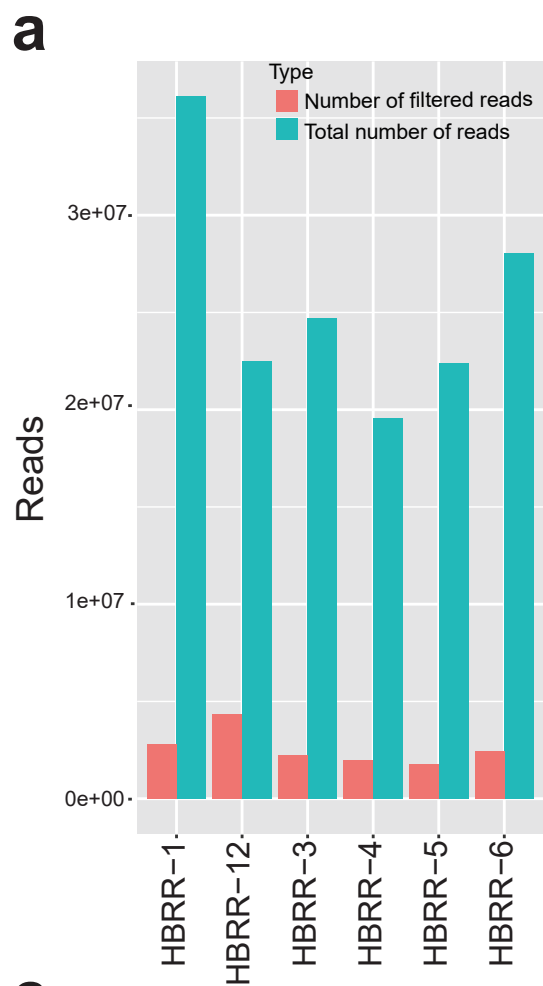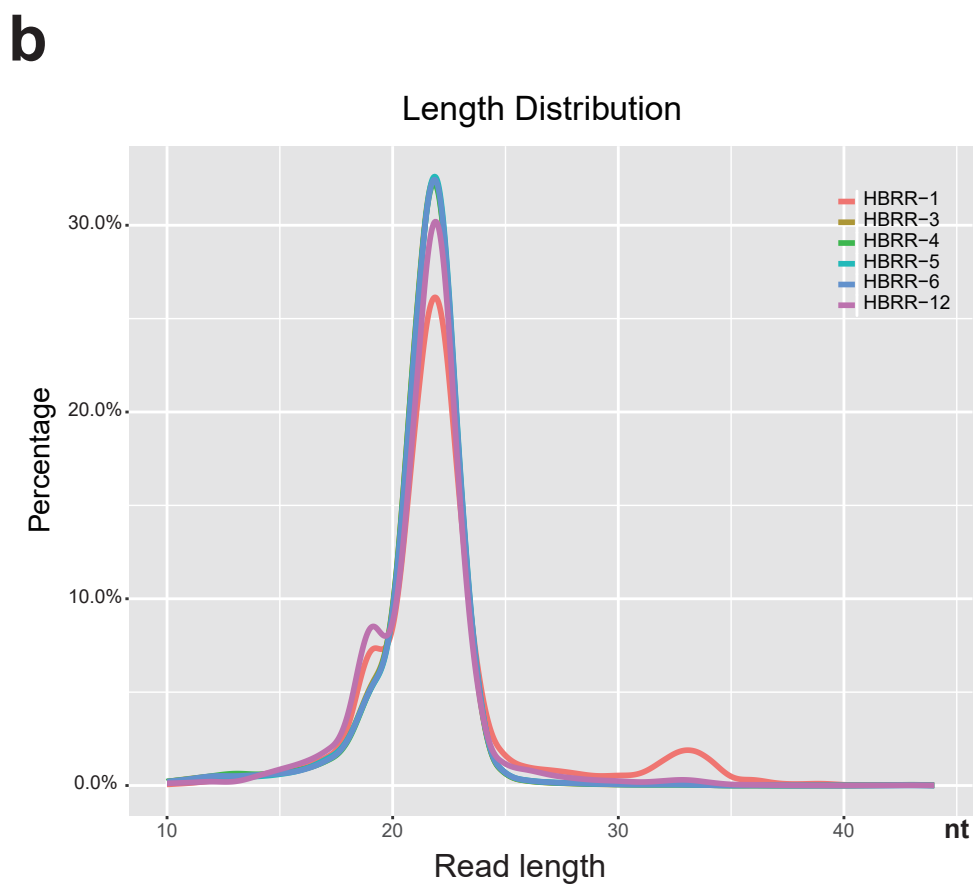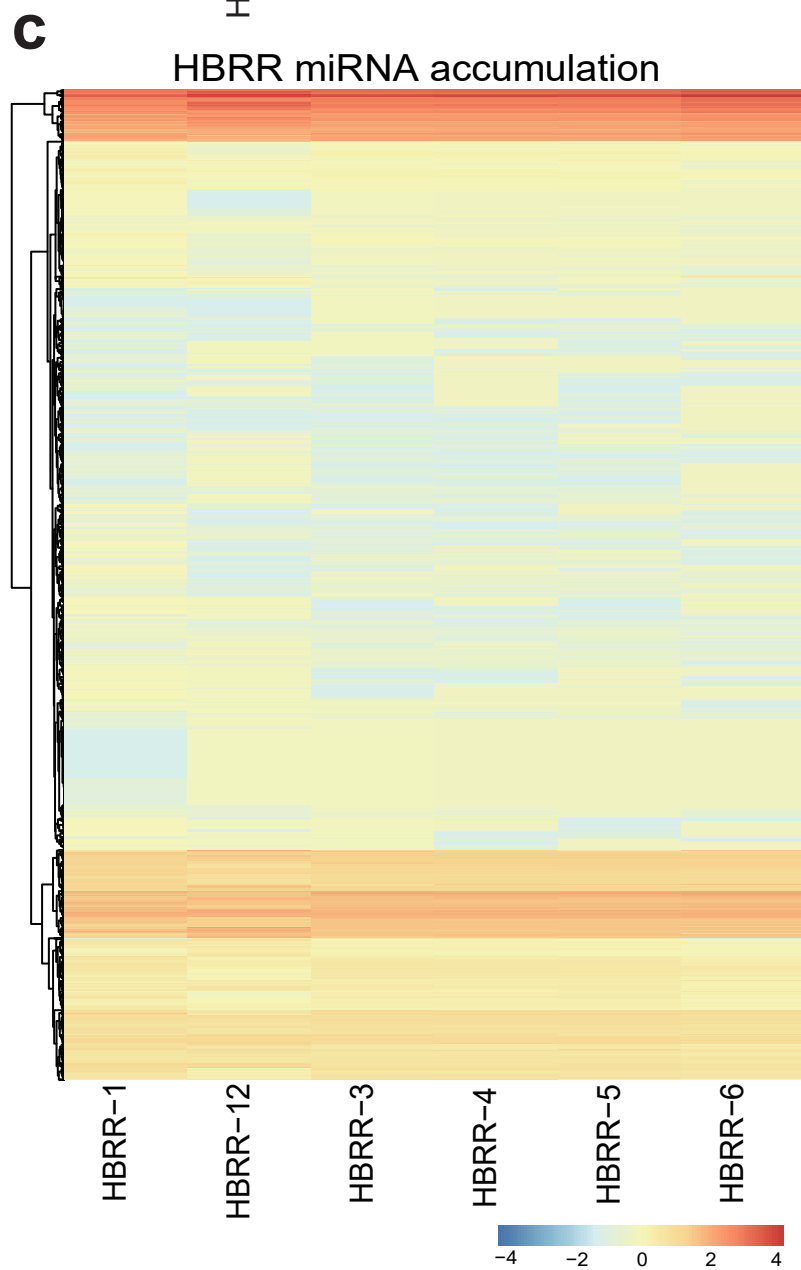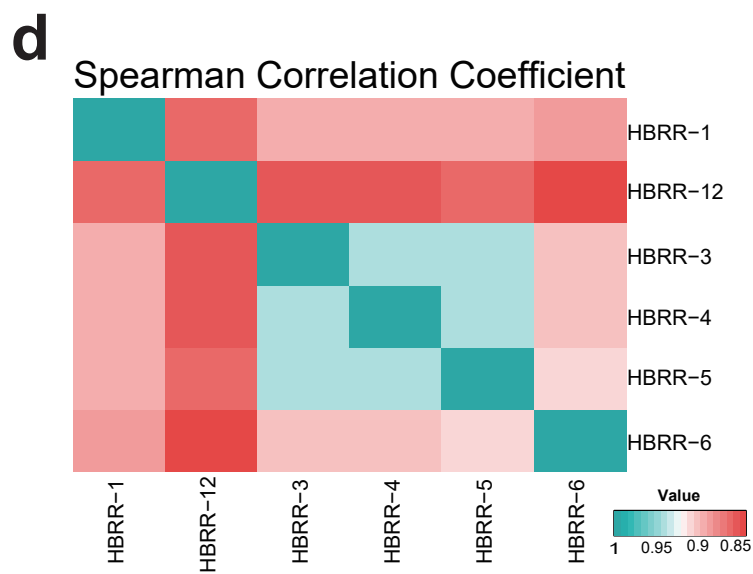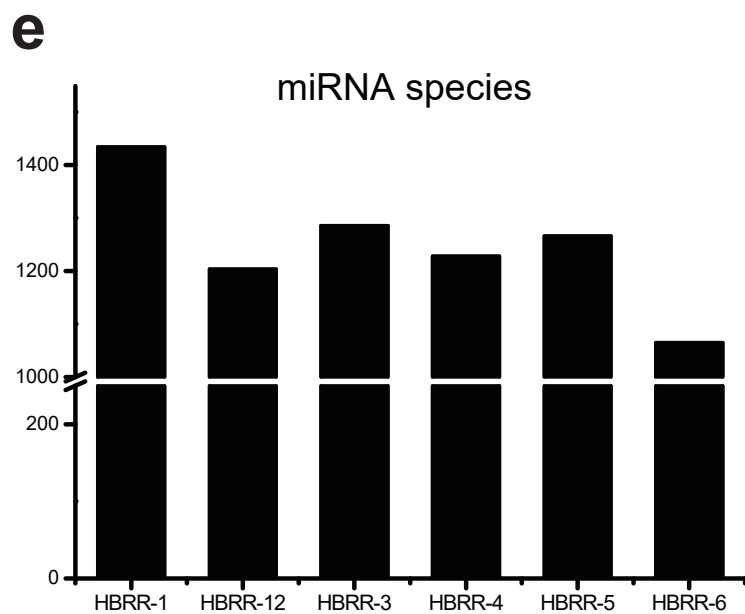

Supplement: FIGURE S4 — HBRR samples showed that batch effect was negligible. [file Data_Sheet_4.PDF]

AD/NC

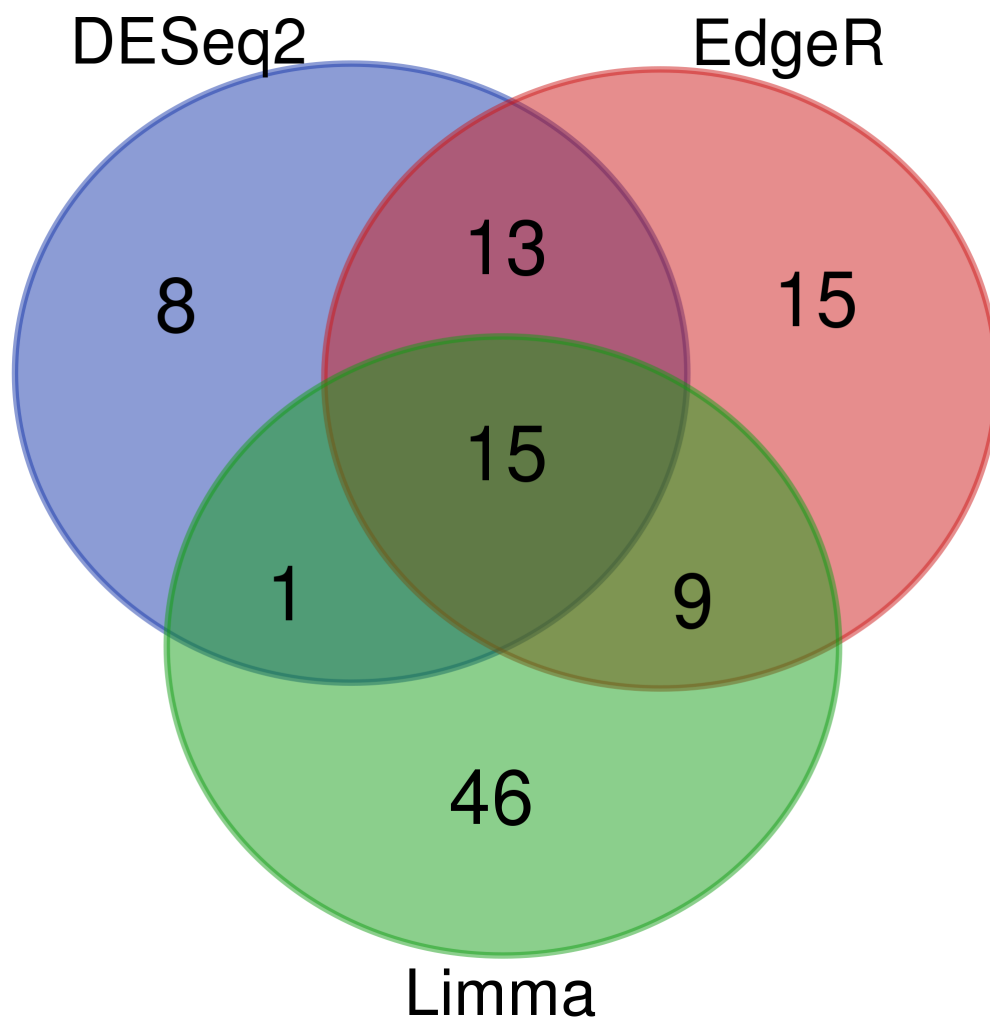

PD/NC

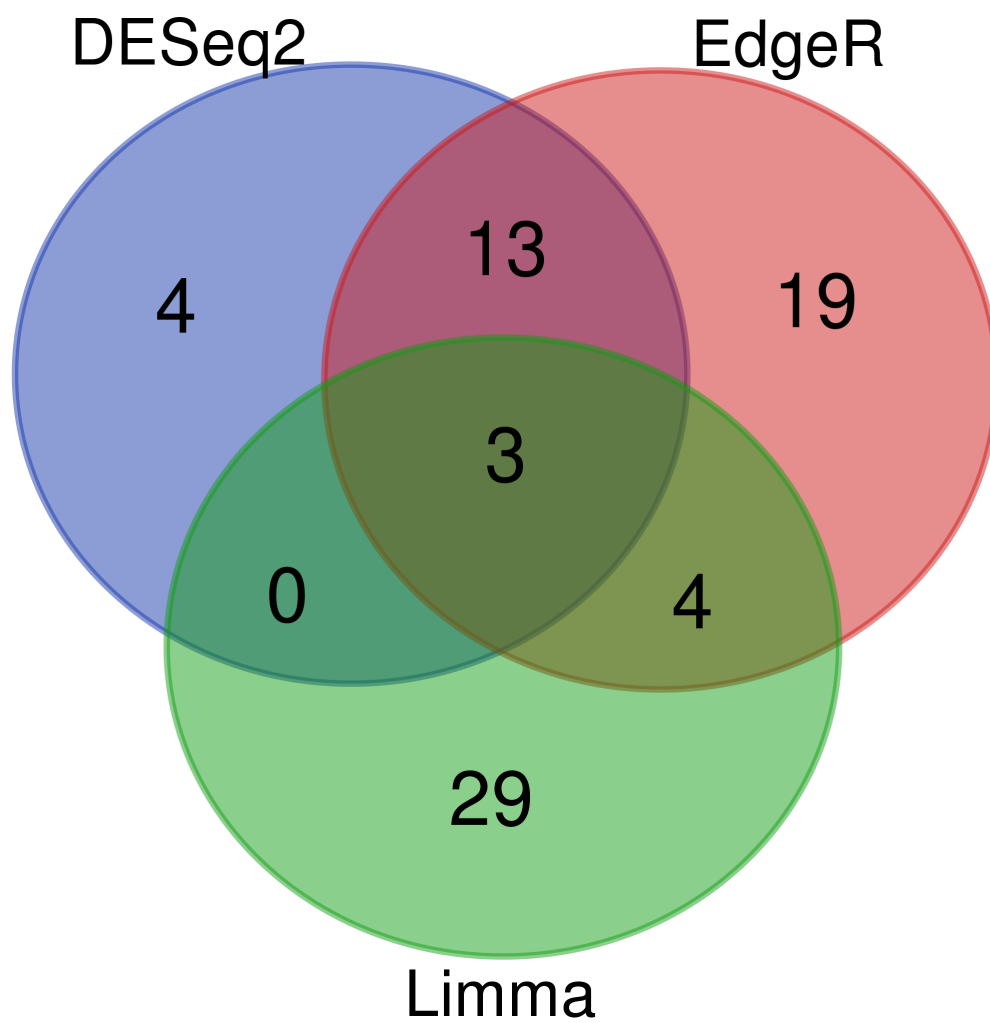

Supplement: FIGURE S5 — Identification of differential miRNA expression. [file Data_Sheet_5.PDF]

# Exosomal sRNA profiles under $-80^{\circ}\text{C}$ storage

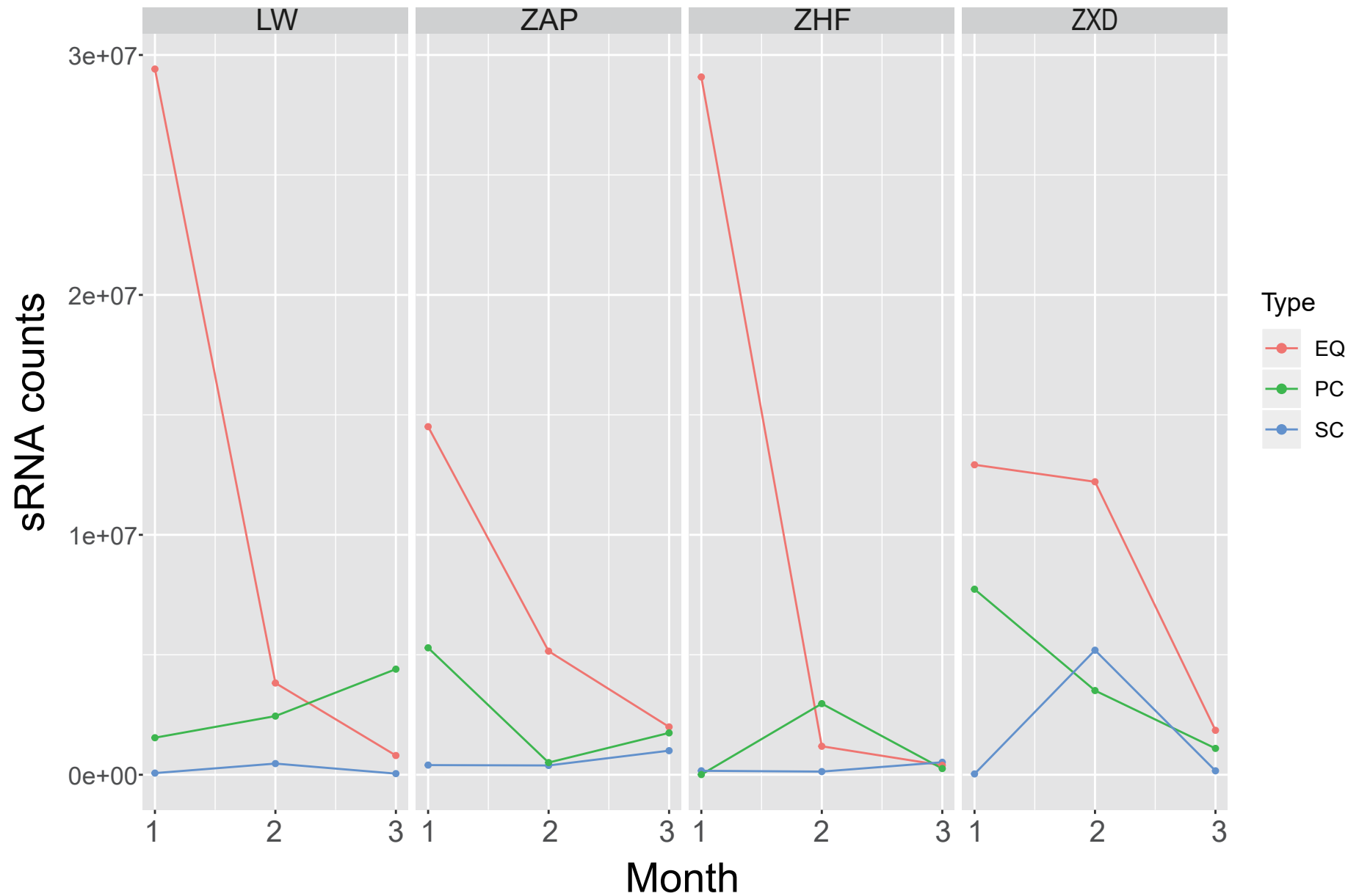

Supplement: FIGURE S6 — Small RNA data quality of samples under -80°C storage. [file Data_Sheet_6.PDF]
